# Glycosylated Natural Products From Marine Microbes

**DOI:** 10.3389/fchem.2019.00879

**Published:** 2020-01-10

**Authors:** Kunlong Li, Jian Cai, Ziqi Su, Bin Yang, Yonghong Liu, Xuefeng Zhou, Jingxia Huang, Huaming Tao

**Affiliations:** ^1^CAS Key Laboratory of Tropical Marine Bio-Resources and Ecology/Guangdong Key Laboratory of Marine Materia Medica, South China Sea Institute of Oceanology, Chinese Academy of Sciences, Guangzhou, China; ^2^College of Earth and Planetary Sciences, University of Chinese Academy of Sciences, Beijing, China; ^3^School of Traditional Chinese Medicine, Southern Medical University, Guangzhou, China; ^4^State Key Laboratory of Ophthalmology, Zhongshan Ophthalmic Center, Sun Yat-sen University, Guangzhou, China

**Keywords:** marine microbes, bacteria, cyanobacteria, fungi, glycosides

## Abstract

A growing body of evidence indicates that glycosylated natural products have become vital platforms for the development of many existing first-line drugs. This review covers 205 new glycosides over the last 22 years (1997–2018), from marine microbes, including bacteria, cyanobacteria, and fungi. Herein, we discuss the structures and biological activities of these compounds, as well as the details of their source organisms.

## Introduction

Sugars are ubiquitous in nature and have a multitude of functions, ranging from serving as a simple source of energy to contributing to molecular-recognition scaffolds that are critical to the interactions/communication among a wide array of biomolecules, cells, tissues, and organisms (Gantt et al., 2011). Not only do sugars work alone in the processes of life, but also they play an important role by combing with secondary metabolites. For instance, glycolipids are carbohydrate-attached lipids, which are widely distributed throughout organisms and involved in the biosynthesis of glycoproteins and serve as ligands for toxins, lectins, bacteria, and viruses. In addition, sugars are also attached to the anomeric carbon of a non-sugar moiety via a glycosidic linkage, such as quinones, lactones, peptides, terpenoids, and alkaloids etc., performed by more than 80 families of glycosyl transferases and those secondary metabolites derive multiple drugs, such as gentamycin, vancomycin, bleomycin, and erythromycin etc. (Grynkiewicz et al., 2008; Yu et al., 2012). Although some glycosides are simply attached to saccharides and saccharide parts in which glycosides are mostly inactive in terms of activity, sometimes they are crucial for overall effects, such as the improvement of a drug's pharmacokinetics and/or dose-limiting toxicities and the improvement for a drug's solubility and selective/non-selective uptake into cells/organs of interest (Gantt et al., 2011; Yu et al., 2012).

**Figure 1:**
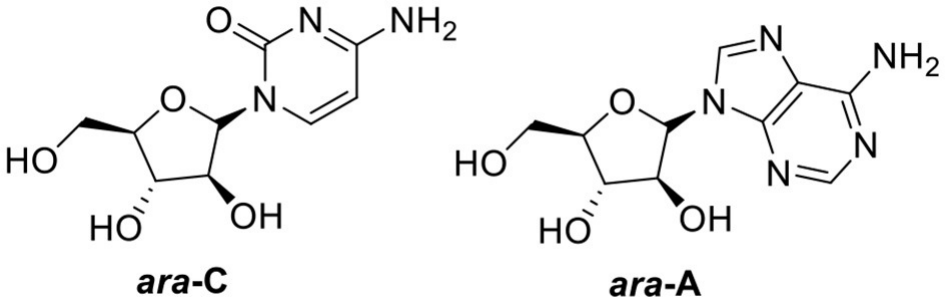
Two FDA-approved nucleosides.

Oceans cover more than 70% of the Earth's surface and host considerable diversity of species. Approximately 30,000 marine natural products had already been identified by the end of 2017 (Jimenez, 2018). The roles of marine natural products in biomedical research and drug development are significant and promising. Many marine natural products have been in clinical stages and the interest in marine natural products is increasing every year (Wang L. et al., 2018). Among these compounds, seven structural types of approved therapeutic agents are considered derivatives of marine natural products, including two nucleosides—the anticancer cytarabine (*ara*-C, FDA-approved in 1969) and the antiviral vidarabine (*ara*-A, FDA-approved in 1976)—derived from two natural arabinonucleosides (Figure 1) (Dyshlovoy and Honecker, 2018). Hence, glycosides have served as a validated platform for the development of many existing front-line drugs (Blanchard and Thorson, 2006).

Given the vital role of glycosides in drug discovery, this review provides a comprehensive overview of the structures and biological activities of 205 glycosides (discovered 1997–2018) from marine-sourced bacteria, cyanobacteria, and fungi, along with the details of their source organisms.

## Discussion

### Marine-Sourced Bacteria Derived Natural Products

#### Quinones

**Figure 2:**
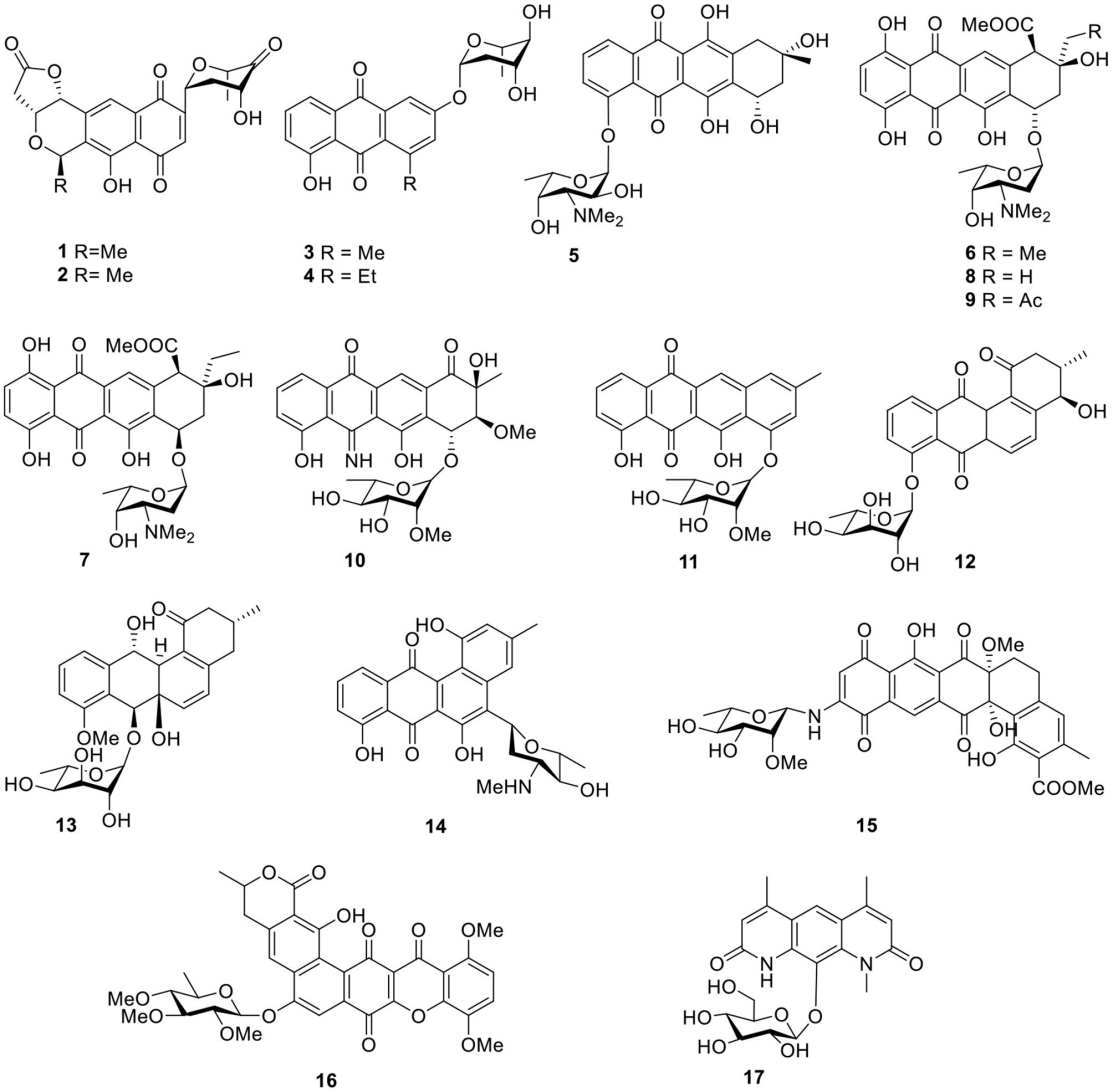
Chemical structures of compounds **1–17** derived from marine-sourced bacteria.

Four quinone-containing metabolites, halawanones A–D (**1–4**, Figure 2), have been isolated from *Streptomycete* sp. BD-18T(41) collected from shallow water sediment. The fraction containing halawanones A–B (**1**–**2**) exhibited inhibitory activity against *Bacillus subtilis* and *Staphylococcus aureus* at 100 μg/disk, but did not inhibit *Escherichia coli* at 100 μg/disk in a disk diffusion assay (Ford et al., 1998).

An anthracycline, komodoquinone A (**5**, Figure 2), with an amino sugar was isolated from the marine *Streptomyces* sp. KS3, derived from marine sediment. Komodoquinone A (**5**) was the first example of all anthracyclines and displayed neurogenic activity against a neuroblastoma cell line (Neuro 2A) at 1 μg/ml (Itoh et al., 2003). Four new anthracycline derivatives—(7S^*^9R^*^10R^*^)-pyrromycin (**6**), (7R^*^9R^*^10R^*^)-pyrromycin (**7**), 1-hydroxyauramycin T (**8**), and 1-hydroxysulfurmycin T (**9**, Figure 2)—were given by a strain *Streptomycete* sp. (CANU Fox 21-2-6) isolated from the mouth of the Fox River. The bioactivity evaluation showed that all four compounds displayed considerable cytotoxicity against P388 cultured cells, with 50% infectious dose (ID_50_) values ranging from 0.4 to 0.06 μg/ml (Phipps et al., 2004). Two anthracyclines, 5-iminoaranciamycin (**10**) and tetracenoquinocin (**11**), were isolated from a culture broth of *Streptomyces* sp. Sp080513GE-26, originally derived from the marine sponge, *Haliclona* sp. Tetracenoquinocin (**11**) exhibited modest cytotoxicity in HeLa and HL-60 cells (IC_50_ 120 and 210 μM, respectively) (Motohashi et al., 2010).

Further chemical investigation of the actinomycete *Saccharothrix espanaensis* An 113, associated with the marine mollusk *Anadara broughtoni*, led to the isolation of two angucyclines saccharothrixmicine A (**12**) and B (**13**, Figure 2). Bioassay results indicated that the saccharothrixmicine-containing fraction exhibited activity toward *Candida albicans* and *Xanthomonas* sp. pv. *Badrii* (Kalinovskaya et al., 2008, 2010). Based on bioassay-guided analyses and the detection of genes encoding for the biosynthesis of secondary metabolites, the marine *Streptomyces* sp. strain HB202, which was isolated from the sponge *Halichondria panacea*, showed profound antibiotic activity and yielded a benz[α]anthracene derivative called mayamycin (**14**, Figure 2). This compound exhibited potent activity against several human cancer cell lines (IC_50_ 0.15–0.33 μM) and inhibited growth of a number of bacteria including antibiotic-resistant strains (IC_50_ 0.31–31.2 μM) (Schneemann et al., 2010).

Arenimycin (**15**, Figure 2) from a strain *Salinispora arenicola* CNR-647 associated with ascidian *Ecteinascidia turbinate* showed significant activity against HCT-116 cells (IC_50_ 1.16 μg/ml). In addition, antibacterial testing with a panel of human Gram-positive pathogens—including various MRSA strains, such as *Enterococcus feacalis* and *Enterococcus faecium*—showed that arenimycin (**15**) exhibited MIC values at or <1 μg/ml (Asolkar et al., 2010). Isolated from the culture broth of *Actinomadura* sp., a polycyclic xanthone, IB-00208 (**16**, Figure 2), showed potent cytotoxic activity against several lines of human and murine tumor cell. Moreover, IB-00208 (**16**) exhibited considerable antibiotic activity against Gram-positive organisms (MIC 0.09–1.4 nM) (Malet-Cascon et al., 2003; Rodriguez et al., 2003). Pseudonocardians C (**17**, Figure 2), a diazaanthraquinone derivative, was produced by the strain SCSIO 01299, which is a marine actinomycete member of the genus *Pseudonocardia* and showed certain *in vitro* cytotoxic activities against the tumor cell lines, SF-268 (human glioma cell line), and MCF-7 (human breast adenocarcinoma cell line) with IC_50_ values of 6.70 and 8.02 μM, respectively (Li S. et al., 2011).

**Figure 3:**
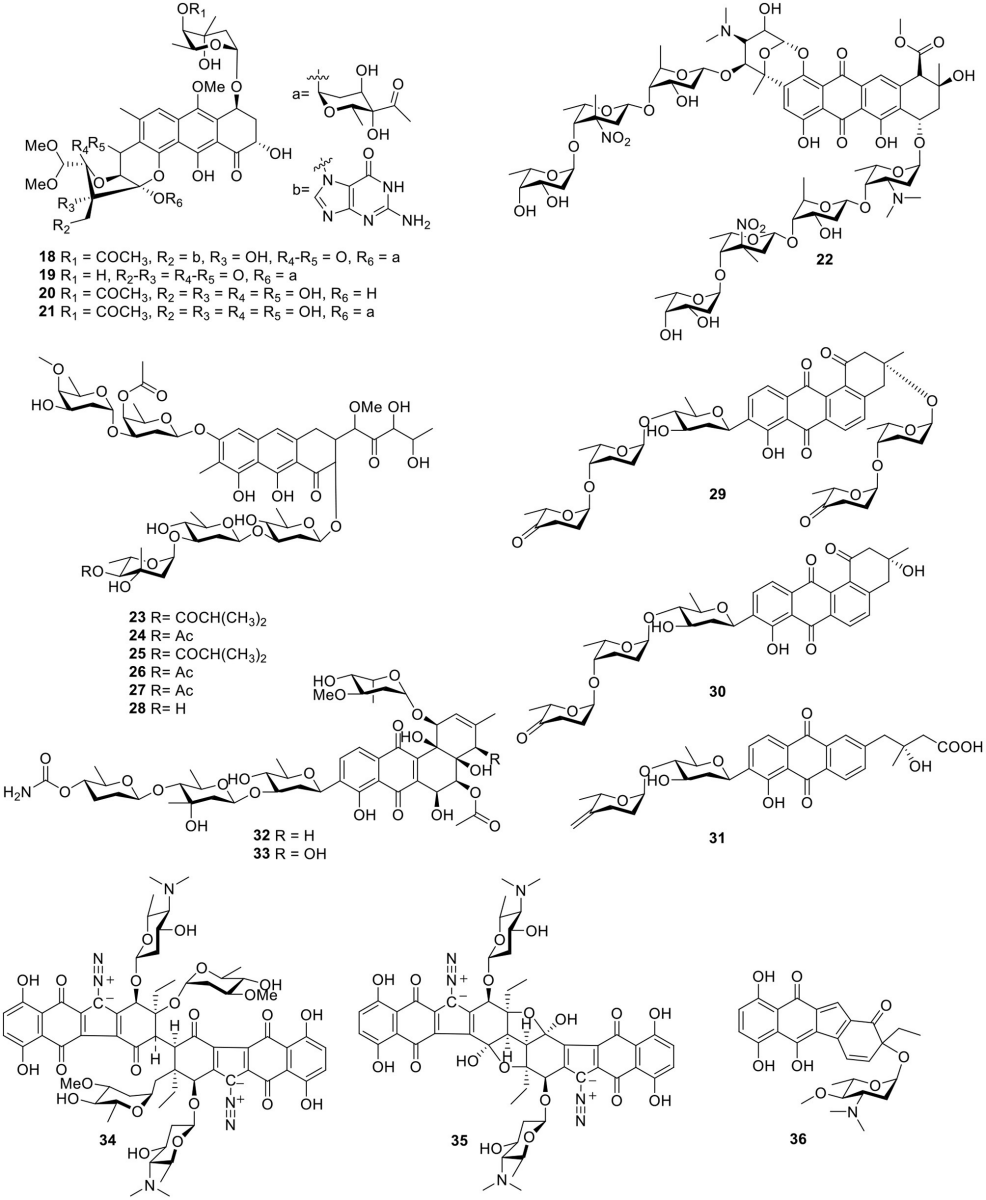
Chemical structures of compounds **18–36** derived from marine-sourced bacteria.

Gutingimycin (**18**, Figure 3) is a natural product with a trioxacarcin skeleton from *Streptomyces* B8652 isolated from a sediment (Maskey et al., 2002, 2004b). The same *Streptomyces* species also yielded trioxacarcins D–F (**19–21**, Figure 3) along with three known trioxacarcins, A–C. Bioactivity tests showed that trioxacarcins A–E exhibited strong antibacterial activity against a range of test organisms—including *B. subtilis, Streptomyces viridoch-romogenes Tu 57, S. aureus*, and *E. coli*—with MIC values of 0.15–2.5 μg/ml, compared with the MIC values >20 μg/ml for gutingimycin. In addition, an experiment against the large-cell lung cancer xenograft LXFL 529 *in vitro* indicated that the activity of trioxacarcin D (**19**) was similar to that previously reported for trioxacarcins A–C, with an IC_50_ value of 0.26 ng/ml (Maskey et al., 2004a). An unattainable bis-nitroglycosylated anthracycline, keyicin (**22**, Figure 3), was produced from coculturing of the producer *Micromonospora* strain with *Rhodococcus*. Biological activity indicated that it inhibited *B. subtilis* and methicillin-sensitive *S. aureus* (MSSA) with MIC values of 8 μg/ml (9.9 μM) and 2 μg/ml (2.5 μM), respectively (Adnani et al., 2017). Six natural products with antibiotic activity—dechromose-A chromomycin A_2_ (**23**), dechromose-A chromomycin A_3_ (**24**), chromomycin A_2_(**25**), chromomycin A_3_(**26**), 4B-*O*-demethylchromomycin (**27**), and chromomycin A_4_ (**28**, Figure 3)—were found from the marine sediment-associated strain *Streptomyces* sp. KMM 9048. Among them, compounds **23** and **24** were established as chromomycin analogs. Antimicrobial activity showed that compounds **25**, **26**, and **27** were mostly active against *B. subtilis* at concentrations of 4.1, 4.2, and 4.3 μM, respectively. In addition, at a concentration of 5 nM, compound **25** exhibited strong inhibition of colony formation of human melanoma RPMI-7951 and SK-Mel-28 cells by 82 and 72%, respectively (Kalinovskaya et al., 2017).

Three angucycline glycosides, designated grincamycins I–K (**29**–**31**, Figure 3), were isolated from the marine-derived actinomycete, *Streptomyces lusitanus* SCSIO LR32. In antitumor tests in five human cancer cells—namely MDA-MB-435, MDA-MB-231, NCI-H460, HCT-116, and HepG2—and human normal breast epithelial cell MCF10A cells, grincamycin J (**30**) showed cytotoxicity with IC_50_ values ranging from 2.6 to 5.4 μM; grincamycin I (**29**) showed strong cytotoxicity against MCF10A with an IC_50_ value of 2.9 μM; however, grincamycin K (**31**) did not exhibit cytotoxic activity. Structure–activity relationships suggested that classical angucyclines and the absence of the disaccharide at 3-*O*-position helped to enhance cytotoxic activity (Lai et al., 2018). With the application of the “Metal Stress” strategy for activating silent gene clusters, the *Streptomyces pratensis* strain NA-ZhouS1, isolated from marine sediment, produced two angucycline antibiotics stremycin A–B (**32–33**, Figure 3). The structures of **32** and **33** showed moderate antibiotic activities with equal MIC values of 16 μg/ml against *Pseudomonas aeruginosa*, MRSA, *Klebsiella pneumonia*, and *Escherchia coli*. In addition, both compounds showed inhibition against *B. subtilis* at an MIC value of around 8–16 μg/ml, respectively (Akhter et al., 2018).

Guided by a biochemical induction assay, two dimeric diazobenzofluorene glycosides, lomaiviticins A–B (**34–35**, Figure 3), were isolated from the halophilic actinomycete *LL*-37I366, which was found to be a new species, *Micromonospora lomaivitiensis*. Both showed potent DNA-damaging activity at a minimum induction concentration ≤0.1 ng/spot and lomaiviticin A (**34**) exhibited cleaved double-stranded DNA under reducing conditions. In an assay against a number of cancer cell lines, lomaiviticin A (**34**) also possessed a unique cytotoxicity profile with IC_50_ values ranging from 0.01 to 98 ng/ml as compared to those of known DNA-damaging drugs, such as adriamycin and mitomycin C. Both lomaiviticins A–B (**34–35**) also exhibited potent antibiotic activity against *S. aureus* and *E. faecium* (He et al., 2001). Continuous searching for benzo[*b*]fluorene led to the discovery of nenestatin A (**36**, Figure 3) produced from the deep sea-derived *Micromonospora echinospora* SCSIO 04089. Comparative bioinformatic analysis has indicated a high similarity of nenestatin A (**36**) and lomaiviticin gene clusters and has led to elucidation of similar biosynthetic pathways, including a conserved set of enzymes for the formation of a diazo group (Jiang X. et al., 2017).

#### Macrocyclic Lactones

**Figure 4:**
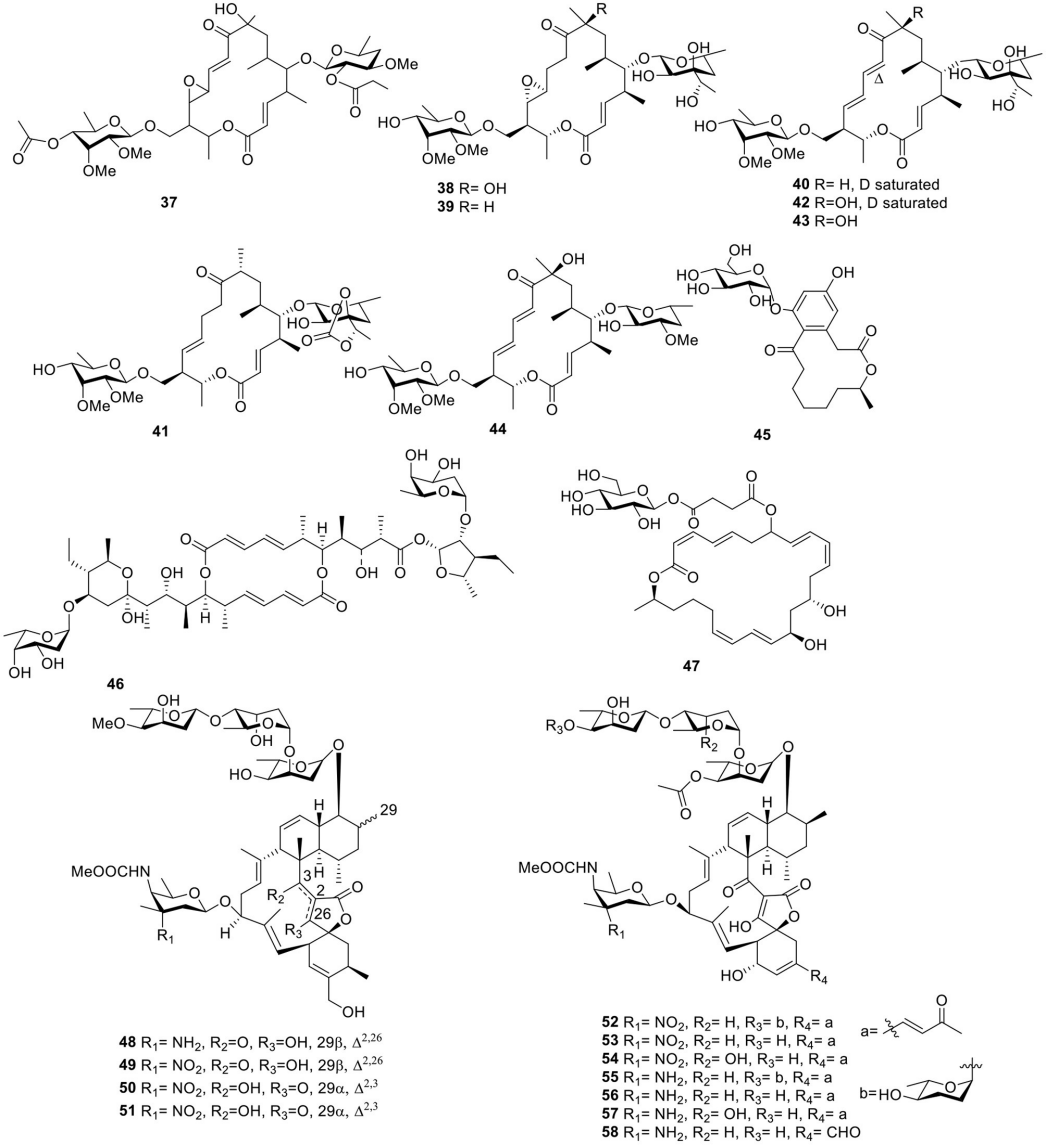
Chemical structures of compounds **37–58** derived from marine-sourced bacteria.

With the application of bioassay-guided analyses, the macrolide antibiotic, chalcomycin B (**37**, Figure 4), was produced by the marine *Streptomycete* sp. B7064 derived from the mangrove sediment. Chalcomycin B (**37**) displayed activity against some microorganisms and microalgae (Asolkar et al., 2002). During the course of searching for bioactive secondary metabolites, the marine microbe *Streptomycetes* sp. strain HK-2006-1 from a marine sediment, produced six 16-membered macrolides, aldgamycins J–O (**38–43**, Figure 4), some of which exhibited strong antibacterial activity against *S. aureus* 209P, such as aldgamycins M–O (**41–43**), which possessed MIC values of 16–32 μg/ml. Structure–activity relationships showed that OH-8 and the double bonds from C-10 to C-13 were beneficial for antibacterial activities (Wang et al., 2016). Further investigation for the *Streptomycetes* sp. strain HK-2006-1 led to a macrolide, chalcomycin E (**44**, Figure 4; Jiang S. et al., 2017). 7-*O*-α-D-glucopyranoside (**45**, Figure 4), a macrolide with a rare α-D-glucopyranose substituent, was isolated from marine actinomycete *Pseudonocardia* sp. HS7, originated from the cloacal aperture of sea cucumber *Holothuria moebii*. A bioassay test indicated that compound **45** displayed modest activity against cancer cell lines with IC_50_ values of 20.84–81.01 μM (Ye et al., 2016). Further investigation for a strain of *Streptomyces hygroscopicus* OUPS-N92 obtained from the marine fish *Halichoeres bleekeri* led to a macrolide, halichoblelide (**46**, Figure 4), with potent cytotoxic activity against the murine P388 cell line and against 39 human cancer cell lines (Yamada et al., 2002). A glycosylated macrolide, macrolactin W (**47**, Figure 4), together with two known macrolides, macrolactins A and Q from the marine *Bacillus* sp. 09ID194 exhibited potent activity against *B. subtilis* (KCTC 1021), *S. aureus* (KCTC 1916), *E. coli* (KCTC 1923), and *P. aeruginosa* (KCTC 2592) with an MIC value of 64 μg/ml (Mondol et al., 2011).

Two derivatives of kijanimicin, lobophorins A–B (**48–49**, Figure 4), were produced from the fermentation broth of the marine actinomycete strain # CNC-837 obtained from the surface of the Caribbean brown alga, *Lobophora variegate*, and both of them exhibited potent antiinflammatory activities in a Phorbol-Myristate-Acetate (PMA)-induced mouse ear edema model (Jiang et al., 1999). Another two analogs, lobophorins C–D (**50–51**, Figure 4), produced by the actinomycete *Streptomyces carnosus* AZS17 obtained from marine sponges *Hymeniacidon* sp. Lobophorin C (**50**), displayed strong cytotoxic activity against the cellular proliferation of 7,402 hepatoma cells with an IC_50_ value of 0.6 μg/ml. In addition, lobophorin D (**51**) had a potent inhibitory effect on the growth of the human breast cancer cell line, MDA-MB 435, with an IC_50_ value of 7.5 μM (Wei et al., 2011). On the basis of bioassays, seven kijanimicin derivatives, microsporanates A–F (**52–57**) and tetrocarcin P (**58**, Figure 4), were isolated from the marine-derived *Micromonospora harpali* SCSIO GJ089. Among them, compounds **52–54** displayed vital growth-inhibiting activities against *B. subtilis* BS01 and *Bacillus thuringiensis* BT01, with MIC values of 0.016–0.5 μg/ml, and compounds **55–58** exhibited moderate activities against *B. subtilis* BS01 and *B. thuringiensis* BT01 with MIC values of 1.0–8.0 μg/ml (Gui et al., 2017).

#### Lipids

**Figure 5:**
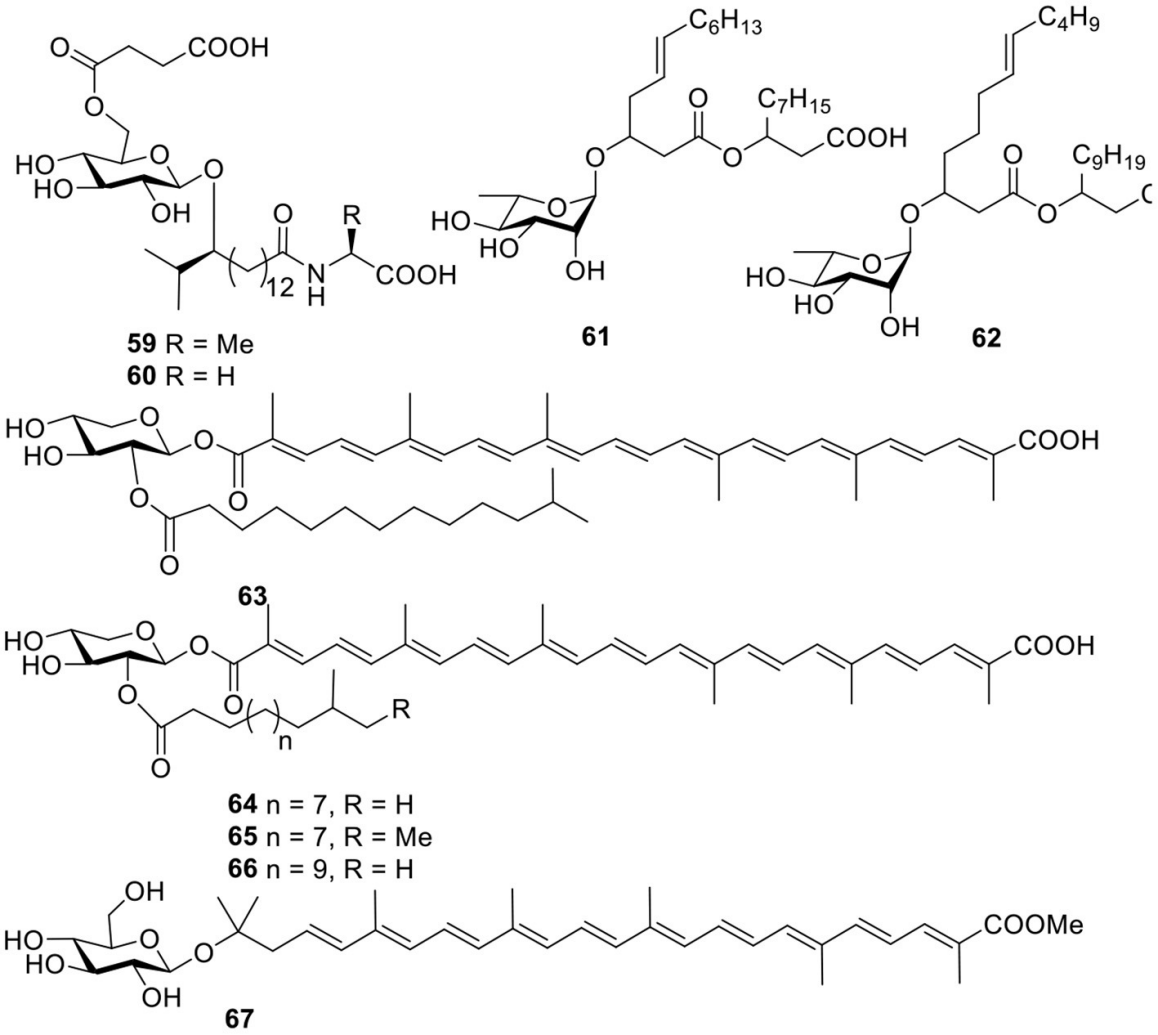
Chemical structures of compounds **59–67** derived from marine-sourced bacteria.

Two unique glycolipopeptides, ieodoglucomides A–B (**59–60**, Figure 5), were produced by the marine-derived bacterium *Bacillus licheniformi* and acted as broad spectrum, moderately active antimicrobial agents. In addition, ieodoglucomide B (**60**) displayed cancer growth inhibition against lung cancer (NCI-H23) and stomach cancer (NUGC-3) cell lines, with GI_50_ values of 25.18 and 17.78 μg/ml, respectively (Tareq et al., 2012). Based on bioassay-guided purification, strain *Pseudomonas* BNT1, isolated from Antarctic sub-sea sediments, produced two rhamnolipids (**61–62**, Figure 5), and in an antibacterial experiment, compound **61** had the lowest MBC values against *Burkholderia cenocepacia* (3.12 μg/ml) and *S. aureus* (3.12 μg/ml) (Tedesco et al., 2016).

#### Terpenoids

A acyl glyco-carotenoic acid, diapolycopenedioic acid xylosyl ester (**63**, Figure 5), was produced from the marine bacterium *Rubritalea squalenifaciens*, belonging to the first subdivision of *Verrucomicrobia* and possessed potent antioxidative activity with an IC_50_ value of 4.6 μM (10.9 μM for β-carotene) (Shindo et al., 2007). Another three analogs, diapolycopenedioic acid xylosyl esters A–C (**64–66**, Figure 5), were obtained from the marine bacterium *R. squalenifaciens*, which was isolated from the marine sponge *Halichondria okadai*. Of the three compounds, compound **64** exhibited ^1^O_2_ suppression activity with an IC_50_ of 5.1 μM (Shindo et al., 2008a). With the same ^1^O_2_ suppression activity as compound **64**, methyl glucosyl-3, 4-dehydro-apo-8′-lycopenoate (**67**, Figure 5) was produced by the marine bacterium *Planococcus maritimu*s strain iso-3 (Shindo et al., 2008b).

#### Alkaloids

**Figure 6:**
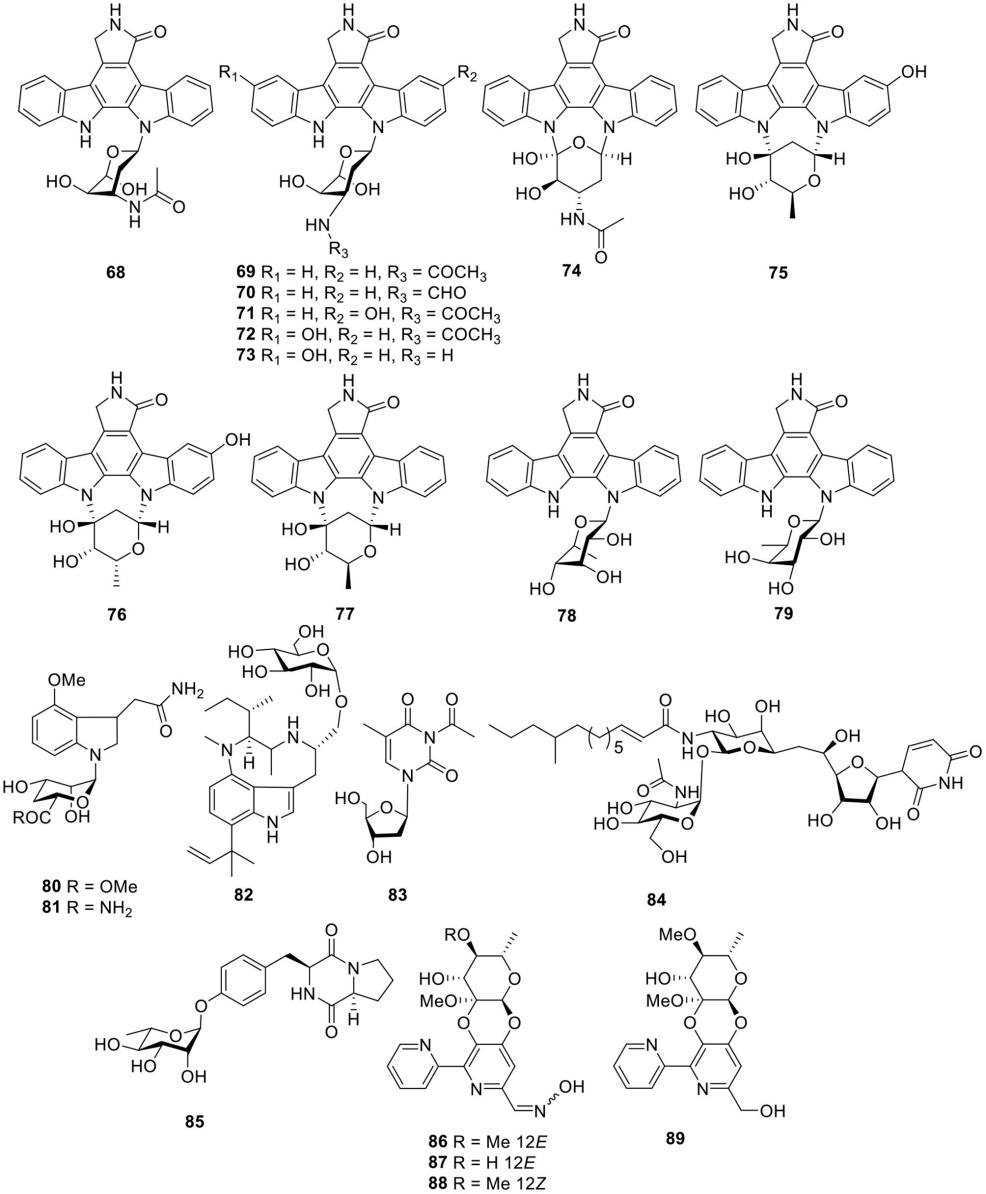
Chemical structures of compounds **68–89** derived from marine-sourced bacteria.

Twelve indolocarbazoles **68–70**, **71–76**, and **77–79** (Figure 6) were isolated from the marine-derived *Streptomyces* sp. A68, *Streptomyces* sp. DT-A61, and *Streptomyces* sp. A65, respectively. Bioactivity testing showed that these indolocarbazoles had cytotoxic activities toward PC-3 cell lines with IC_50_ values of 0.8–41.3 μM. In addition, most of these indolocarbazoles also showed potent kinase inhibitory activities against protein kinase C alpha (PKCα), Roh associated protein kinase 2 (ROCK2), Bruton's tyrosine kinase (BTK), and apoptosis signal-regulating kinase 1 (AKS1). For instance, compound **7** displayed a notable inhibitory effect against ROCK2 with an IC_50_ value of 5.7 nM, which was similar to that of the positive control, staurosporine (IC_50_ = 7.8 nM). Structure–activity relationships for this set of indolocarbazoles suggested that when the sugar, connected with the K252c unit, was similar to that of staurosporine, the compound would be more effective than those without sugar moiety or those with only a single attachment of the sugar to the aromatic aglycone (Qin et al., 2018; Wang J. N. et al., 2018; Zhou et al., 2018).

Two rare N-glycosyl indoles, kahakamides A–B (**80–81**, Figure 6), were given by the actinomycete *Nocardiopsis dassonvillei*, isolated from a shallow water sediment sample. Bioassay testing indicated that kahakamide A (**80**) showed slight inhibition of *B. subtilis* in a disc-diffusion assay (Schumacher et al., 2001). From an actinomycete belonging to the family *Nocardiopsaceae, Marinactinospora thermotolerans* SCSIO 00652, methylpendolmycin-14-*O*-α-glucoside (**82**, Figure 6) was identified and found to exhibit antiplasmodial activities against the *Plasmodium falciparum* lines Dd2 and 3D7 with IC_50_ values of 5.03 and 10.43 μM, respectively (Huang et al., 2011). One deoxyuridine (**83**, Figure 6), obtained from the *Streptomyces microflavus* strain, No. HVG29, represents the first example of acetyl deoxyuridine from marine-derived actinomycetes which was isolated from the marine sponge, *Hymeniacidon perlevis* (Li K. et al., 2011). Tunicamycin E (**84**, Figure 6), a new natural nucleoside antibiotic, was isolated from marine-derived *Streptomyces xinghaiensis* SCSIO S15077 and exhibited moderate antifungal activity against *B. thuringiensis, B. thuringiensis*, and *C. albicans* with MIC values of 2.0, 0.5, and 8.0 μg/ml, respectively (Zhang et al., 2018).

A diketopiperazine glycoside, maculosin-*O*-α-L-rhamnopyranoside (**85**, Figure 6), was obtained from a culture of the marine-derived actinomycete *Streptomyces* sp. ZZ446. Bioassay testing showed that compound **85** displayed antimicrobial activity against methicillin-resistant *S. aureus, E. coli*, and *C. albicans* with MIC values of 27.0–37.0 μg/ml (Chen et al., 2018). Cyanogrisides A–D (**86–89**, Figure 6) were four bipyridine cyclic glycosides from the actinomycete *Actinoalloteichus cyanogriseus* WH1-2216-6. Cyanogrisides A (**86**) and C (**88**) were moderately cytotoxic to three multidrug resistant (MDR) and drug-sensitive parental cell lines, and cyanogrisides B (**87**) reversed the multidrug resistance of K562/A02,MCF-7/Adr, and KB/VCR cells at a concentration of 10 μM, with reversal-fold values of 1.7, 1.2, and 3.6, respectively (Fu et al., 2011).

#### Peptides

**Figure 7:**
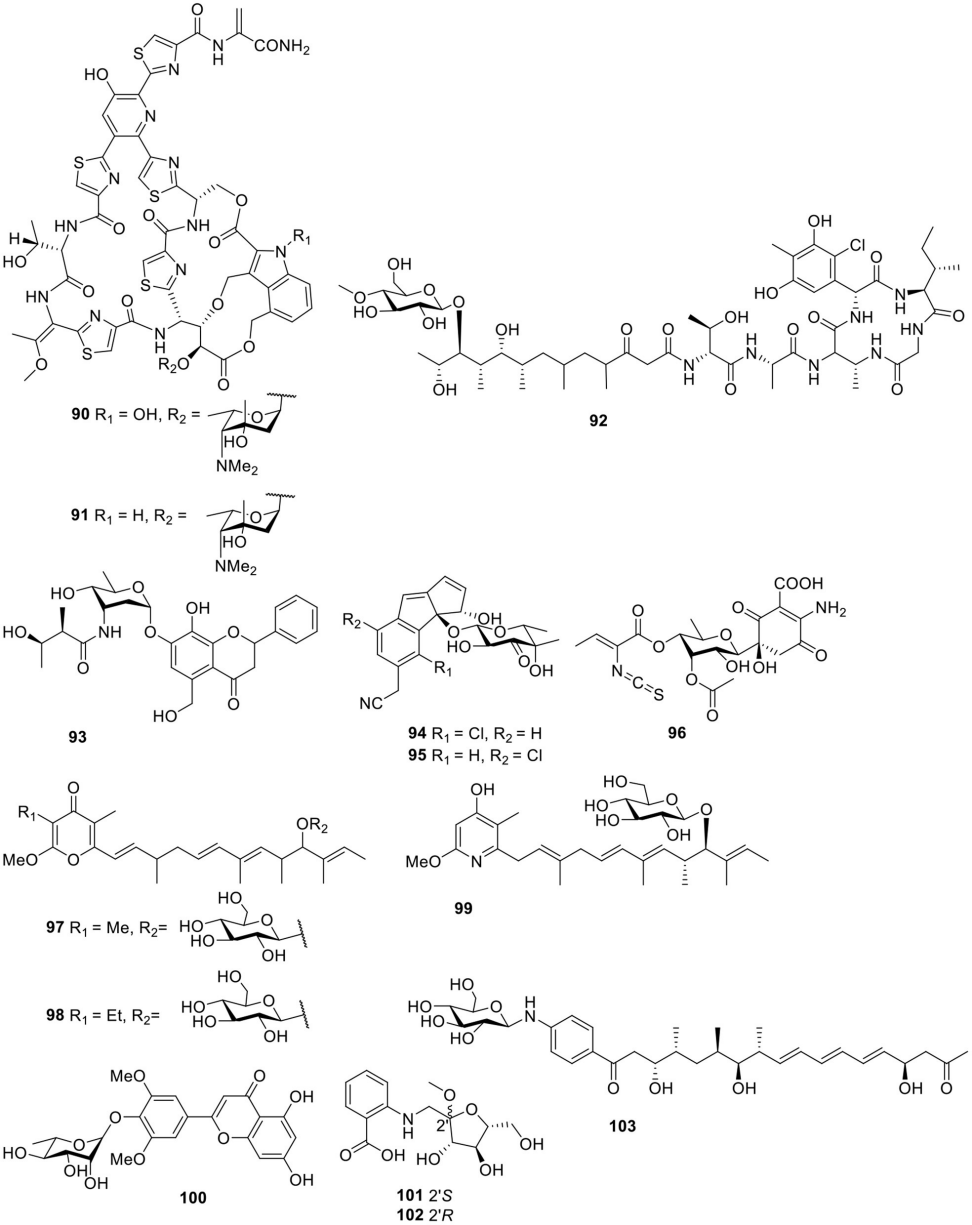
Chemical structures of compounds **90–103** derived from marine-sourced bacteria.

Based on a multi-drug resistant *E. faecium* (MREF) assay, two thiazolyl peptide glycosides, nocathiacins I–II (**90–91**, Figure 7), were isolated from the cultured broth of *Nocardia* sp. WW-12651 (ATCC 202099). The nocathiacins exhibited strong *in vitro* activity against a broad spectrum of Gram-positive bacteria, with MIC values of 0.1–60 ng/ml. In addition, they also showed good *in vivo* efficacy in a systemic *S. aureus* infection mouse model (Leet et al., 2003; Li et al., 2003). One peptide-polyketide glycoside totopotensamide A (**92**, Figure 7) produced by a *Streptomyces* sp. 1053U.I.1a.1b, cultivated from the gastropod mollusk *Lienardia totopotens*, contains a previously undescribed 2,3-diaminobutyric acid-containing macrolactam and an amino acid, 4-chloro-5,7-di- hydroxy-6-methylphenylglycin (Lin et al., 2012).

#### Other Classes

A flavonoid-like glycoside, actinoflavoside (**93**, Figure 7), was detected in the culture broth of the marine *Streptomyces* sp. CNB-689 and showed only weak antibacterial activity against Gram-positive bacteria (Jiang et al., 1997). Further biological and chemical investigation of *Salinispora* strains led to the discovery of a third species with this genus, *Salinispora pacifica*, which produced two chlorinated cyclopenta[α]indene glycosides, cyanosporasides A–B (**94–95**, Figure 7; Oh et al., 2006). Isolated from the marine actinomycetes *Micromonospora matsumotoense* M-412, the first compound of the paulomycin family lacking the paulomycose structure, paulomycin G (**96**, Figure 7), displayed excellent cytotoxic activities against several human tumor cell lines—such as MiaPaca_2, MCF-7, and HepG2—with IC_50_ values of 2.70, 1.58, and 4.30 μM, respectively (Sarmiento-Vizcaino et al., 2017).

Two antitumor pyranone glycosides, PM050511 (**97**) and PM0060431 (**98**, Figure 7), along with their aglycones PM050463 and PM060054, were obtained from the marine-derived *Streptomyces albus*, POR-04-15-053. Bioassay testing suggested that compounds **97–98** showed excellent cytotoxicity against three human tumor cell lines with GI_50_ values in the range of 0.24–2.69 μM (Schleissner et al., 2011). A cytotoxic piericidin derivative, glucopiericidin C (**99**, Figure 7), was isolated from the marine-derived *Streptomyces* species B8112 and showed a concentration-dependent cytotoxicity toward a panel of 36 human tumor cell lines with an IC_50_ value of 2.0 μM (mean IC_70_ = 4.2 μM), in addition to the same antibacterial activity as glucopiericidin A (Shaaban et al., 2011). One flavonoid derivative, flavoside A (**100**, Figure 7), was produced from the EtOAc extract of the culture broth of the sea urchin (*Anthocidaris crassispina*)-derived actinobacterium, *Streptomyces* sp. HD01 (Guo et al., 2019). According to the HPLC-UV profile, the *Streptomyces* sp. CMN-62 isolated from an unidentified sponge sample was selected for its chemical investigation and produced two anthranilate-containing alkaloids, anthranosides A–B (**101–102**, Figure 7; Che et al., 2018). Chemical analysis of these actinomycete strains using LC/MS identified a *Streptomyces* sp. SNM31 and led to a metabolite, mohangic acid E (**103**, Figure 7), which was the first glycosylated compound discovered in the *p*-aminoacetophenonic acid family and exhibited good quinone-reductase induction activity at a concentration of 20 μM (Bae et al., 2016).

### Marine-Sourced Cyanobacteria Derived Natural Products

#### Macrocyclic Lactones

**Figure 8:**
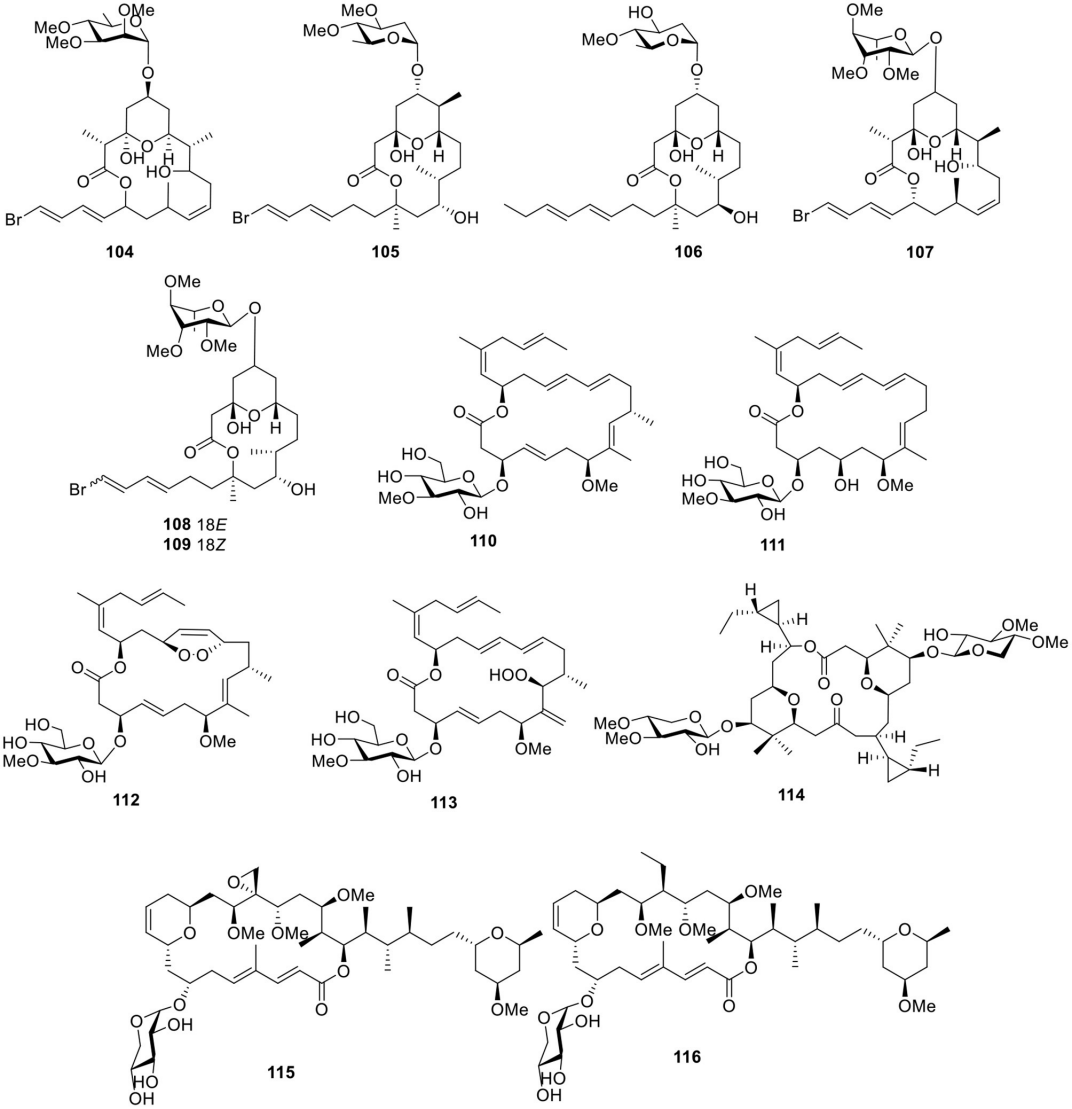
Chemical structures of compounds **104–116** derived from marine-sourced cyanobacteria.

*Lyngbya bouillonii* Hoffmann and Demoulin is a “superproducer,” which is a filamentous, non-heterocystous, blue-green alga up to 50 μm wide (Klein et al., 1997). Guided by cancer viability assays and the aid of LC-MS, six macrolide glycosides—lyngbyaloside (**104**), lyngbyaloside B (**105**), lyngbouilloside (**106**), 2-epi-lyngbyaloside (**107**), and the regioisomeric 18*E*- and 18*Z*-lyngbyalosides C (**108-109**, Figure 8)—were isolated from *L. bouillonii*. Bioassay testing suggested that lyngbyaloside B (**105**) exhibited weak cytotoxicity against KB cells, with an IC_50_ value of 4.3 μM and showed a smaller effect on LoVo cells (IC_50_ ≈ 15 μM); additionally, lyngbouilloside (**106**) was only moderately cytotoxic to neuro-2a neuroblastoma cells (IC_50_ = 17 μM) (Klein et al., 1997; Luesch et al., 2002; Tan et al., 2002; Matthew et al., 2010).

Bioassay-guided investigation of the marine cyanobacterium *Lyngbya* sp., collected in Okinawa Prefecture, led to an 18-membered macrolide glycoside, biselyngbyaside (**110**, Figure 8). Biselyngbyaside (**110**) exhibited broad-spectrum cytotoxicity in a panel of human tumor cell lines and likely inhibited cancer cell proliferation through a mechanism indicated by COMPARE analyses (Teruya et al., 2009). Chemical investigation of the marine cyanobacterium *Lyngbya* sp., collected from the Tokunoshima Island, Japan, led to three new analogs of biselyngbyaside (**110**), biselyngbyasides B–D (**111–113**, Figure 8). Biselyngbyaside B (**111**) was shown to induce apoptosis in HeLa S_3_ cells and HL60 cells. Further investigation of this activity in HeLa S_3_ cells indicated that apoptosis is likely mediated through increasing cytosolic Ca^2+^ concentrations (Morita et al., 2012).

The dimeric macrolide xylopyranoside, cocosolide (**114**, Figure 8), was obtained from the marine cyanobacterium preliminarily identified as *Symploca* sp. and reduced IL-2 production without significantly affecting cell viability. Comparison of the activities of analogs indicated the importance of sugars and dimeric structures to the target recognition and engagement process (Gunasekera et al., 2016). Bioassay-guided fractionation of the extract of *Leptolyngbya* sp., collected from the coast of Itoman City in the Okinawa Prefecture (Japan), led to the separation of two macrolactones, leptolyngbyolides A–B (**115–116**, Figure 8), both of which showed strong growth inhibition against HeLa S_3_ cells with IC_50_ values of 0.1 and 0.16 μM, respectively. In addition, structure–activity relationships suggested that the sugar moiety did not affect growth-inhibitory activity (Cui et al., 2017).

**Figure 9:**
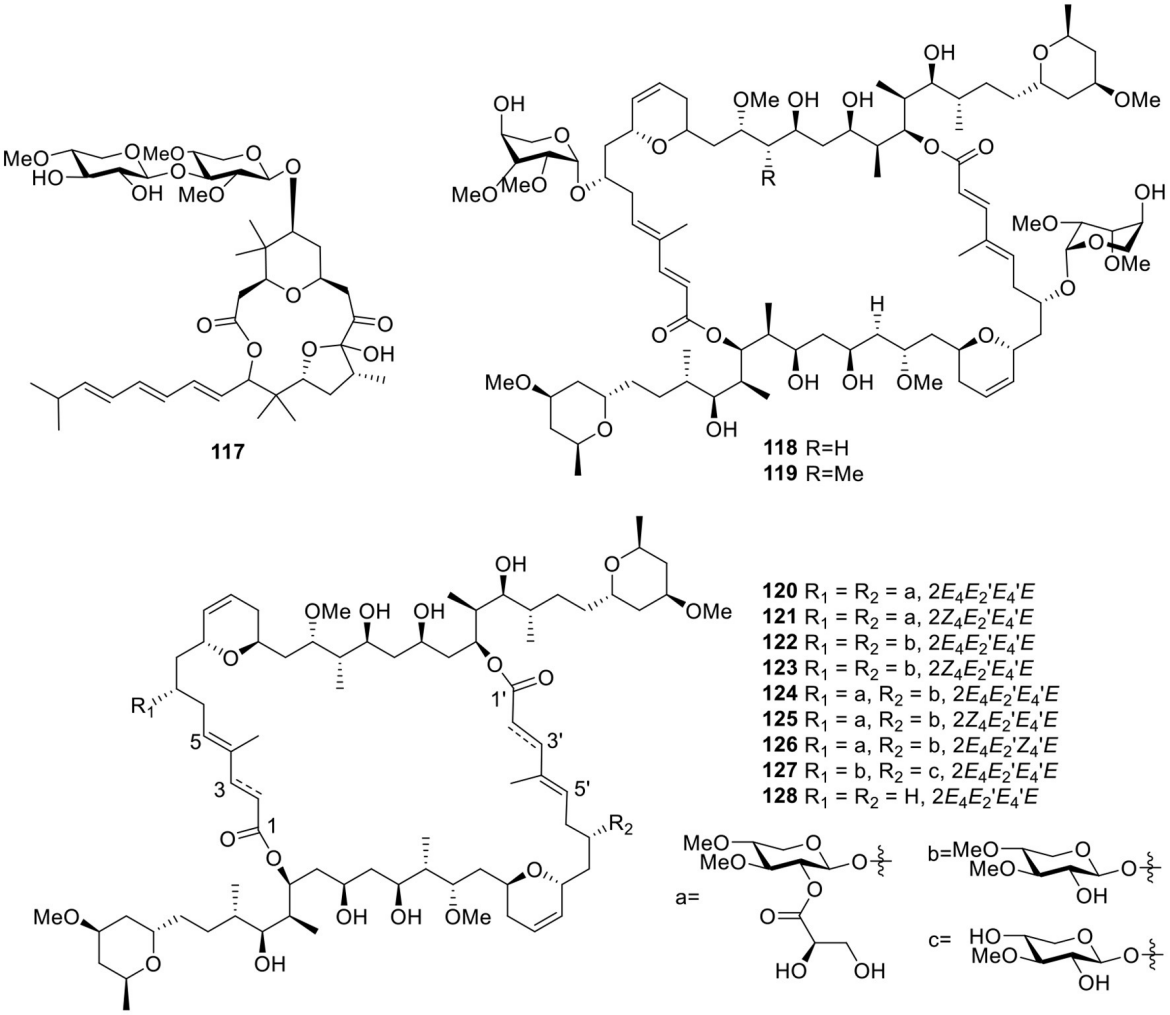
Chemical structures of compounds **117–128** derived from marine-sourced cyanobacteria.

The polycavernoside analog, polycavernoside D (**117**, Figure 9), was isolated from a red-colored *Okeania* sp. and had moderate activity against the human lung carcinoma cell line H-460 (EC_50_ = 2.5 μM). Importantly, polycavernoside D (**117**) was obtained from the Atlantic, whereas polycavernosides previously isolated were derived from the Western Pacific, suggesting that these toxins occur over a much wider geographical range than originally thought (Navarro et al., 2015). Two glycosylated swinholides, ankaraholides A–B (**118**–**119**, Figure 9), were produced by the cyanobacterium, *Geitlerinema* sp., from a Madagascar field collection. Bioassay testing indicated that ankaraholide A (**118**) inhibited proliferation (IC_50_ values) in NCI-H460 (119 nM), Neuro-2a (262 nM), and MDA-MB-435 (8.9 nM) cell lines (Andrianasolo et al., 2005). Under bioassay-guided separation in combination with the MS2-based molecular-networking dereplication tool, nine glycosylated swinholide-type compounds, samholides A–I (**120**–**128**, Figure 9), were separated from the American Samoan marine cyanobacterium cf. *Phormidium* sp. All of these samholides showed potential activities against the human lung cancer cell line H-460 with IC_50_ values ranging from 170 to 910 nM. Comparison of the activities of these samholides suggested that the sugar and glyceric-acid units played important roles in enhancing the cytotoxic activity (Tao et al., 2018).

#### Lipids

**Figure 10:**
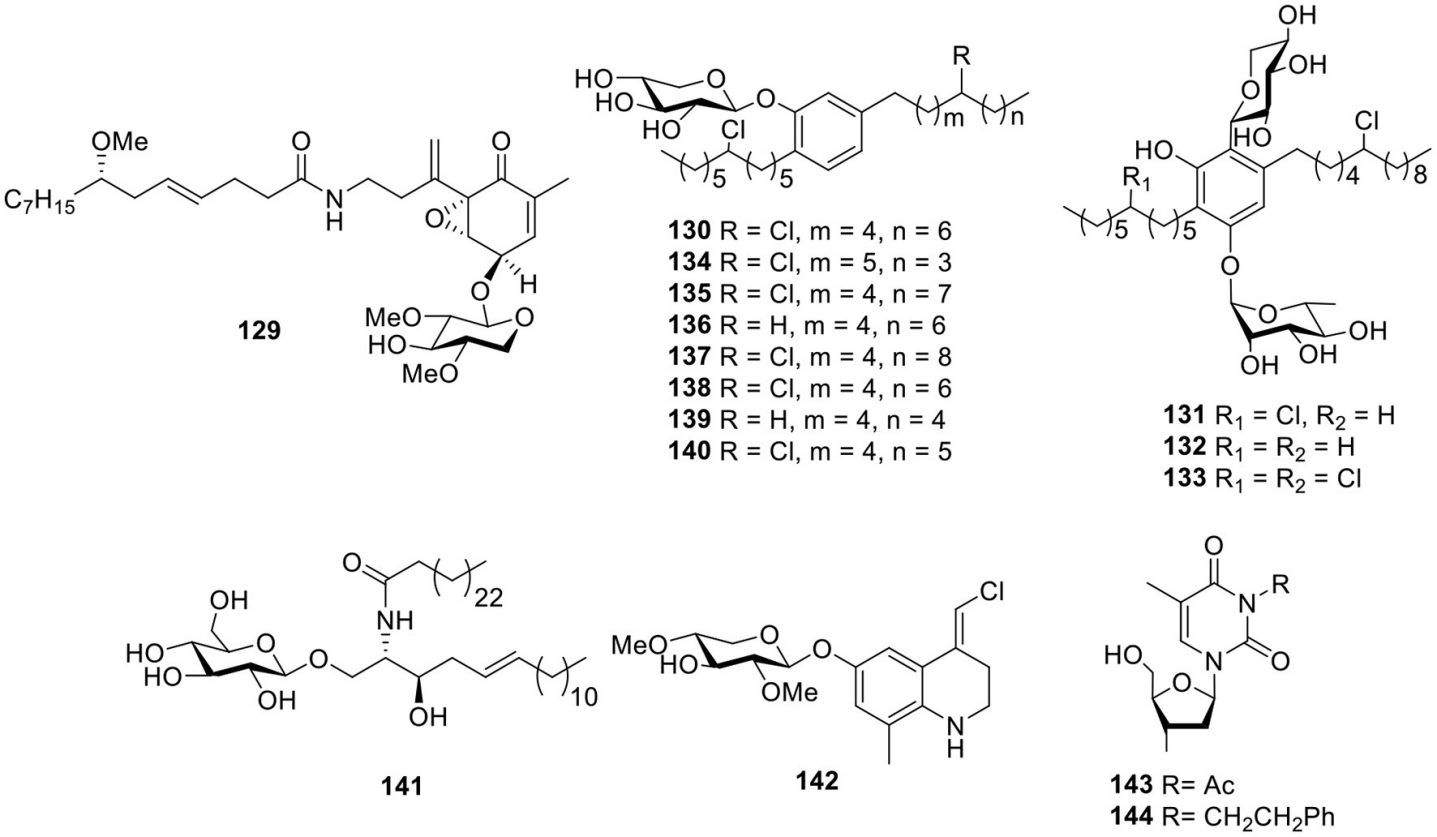
Chemical structures of compounds **129–144** derived from marine-sourced cyanobacteria.

Further investigation of the marine cyanobacterium *Lyngbya majuscula* from Curafao yielded one glycoside metabolite, malynsamide J (**129**, Figure 10). Bioassay testing suggested that malynsamide J (**129**) was toxic to both brine shrimp and fish (Wu et al., 1997). Bartolosides A–D (**130**–**133**, Figure 10), which are unique glycolipids featuring aliphatic chains with chlorine substituents and C-glycosyl parts, were isolated from the filamentous cyanobacterium *Nodosilinea* sp. LEGE 06102 and *Synecho-cystis salina* LEGE 06155, respectively. The determination of the planar structure of bartolosides through key pathway intermediates illustrates the importance of genomics for structure elucidation. In addition, the biosynthesis of the diglycosylated dialkylresorcinol skeleton of bartolosides B–D (**131**–**133**) involves first the head-to-head condensation of an α, β-unsaturated fatty acyl-ACP thioester with a β-keto-fatty acyl-ACP thioester, catalyzed by the ketosynthase, BrtD (Leao et al., 2015). Another seven analogs, bartolosides E–K (**134**–**140**, Figure 10), produced from *Synechocystis salina* LEGE 06099, a strain closely related to the *Synecho-cystis salina* LEGE 06155 and bartoloside E (**134**), showed antitumor activities against the MG-63, RKO, and T-47D cell lines with IC_50_ values of 39, 40, and 22 μM, respectively (Afonso et al., 2016). One cerebroside, mooreaside A (**141**, Figure 10), was produced by the marine cyanobacterium *Moorea producens*, collected from the Red Sea and displayed moderate activity toward the MCF-7 cancer cell line with an IC_50_ value of 20.5 μM (Youssef et al., 2016).

#### Alkaloids

Chemical investigation of the crude organic extract of *L. majuscula* from Puerto Rico resulted in the quinoline alkaloid **142** (Figure 10), the geometry of which was established as (*E*) by ^1^H-^13^C coupling constant measurements from HSQMBC NMR experiments (Nogle and Gerwick, 2003). Except for the mooreaside A (**141**), the marine cyanobacterium *M. producens* also yielded two nucleoside derivatives, 3-acetyl-2′-deoxyuridine (**143**) and 3-phenylethyl-2′-deoxyuridine (**144**, Figure 10), both of which showed moderate activity toward the MCF-7 cancer cell line with IC_50_ values of 18.2 and 22.8 μM, respectively (Youssef et al., 2016).

### Marine-Sourced Fungi Derived Natural Products

#### Quinones

**Figure 11:**
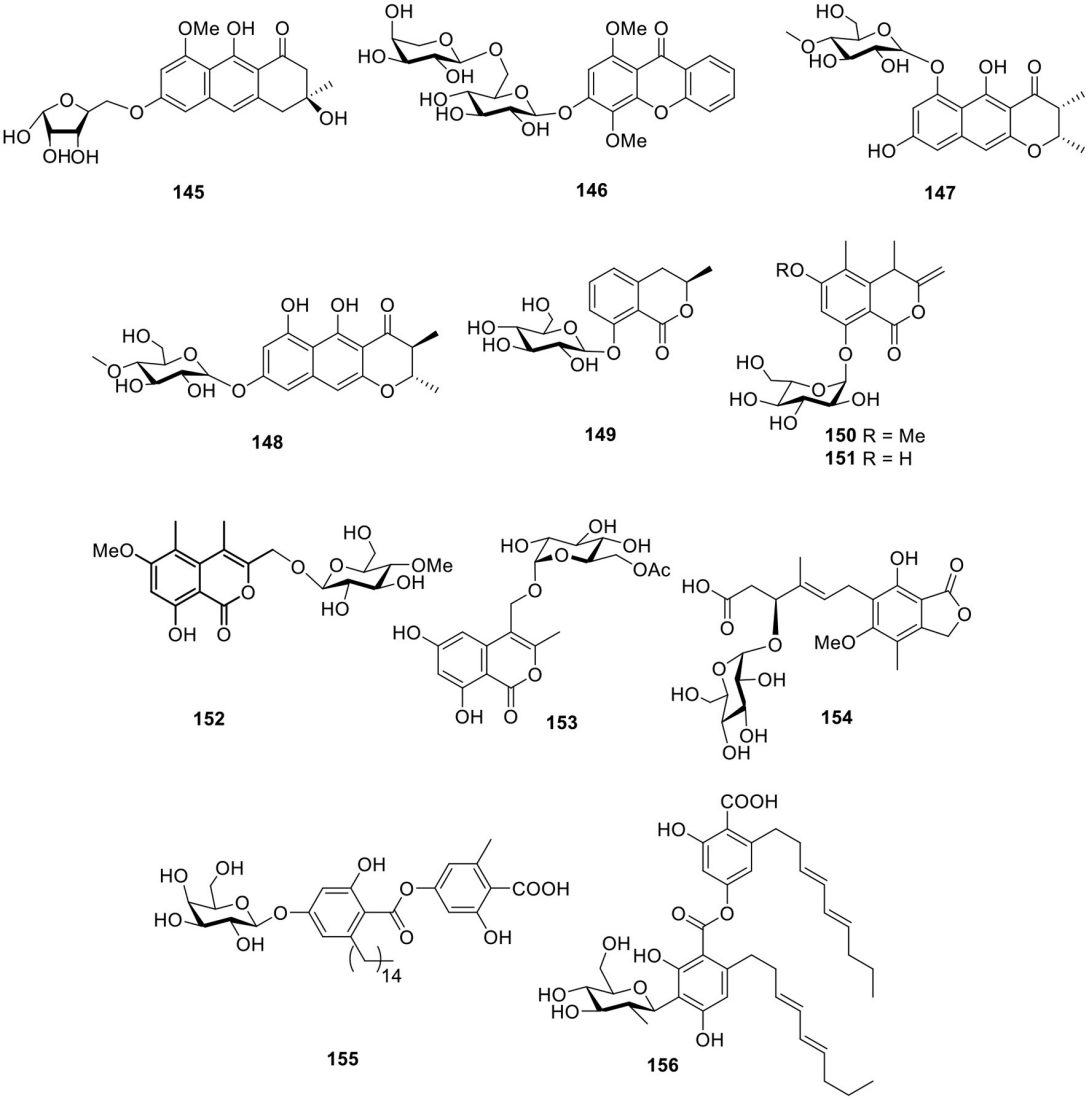
Chemical structures of compounds **145–156** derived from marine-sourced fungi.

A anthracene glycoside, asperflavin ribofuranoside (**145**, Figure 11), was isolated from the marine-derived fungus *Microsporum* sp. Compound **145** showed radical scavenging activity against DPPH with an IC_50_ value of 14.2 μM and also exhibited moderate antibacterial activity against the methicillin-resistant and multidrug-resistant *S. aureus* with an MIC value of 50.0 μg/ml (Li et al., 2006). The xanthone *O*-glycoside, 3-*O*-(6-*O*-α-L-arabinopyranosyl)-β-D-gluco- pyranosyl-1,4-dimethoxyxanthone (**146**, Figure 11), was obtained from the mangrove endophytic fungus *Phomopsis* sp. ZH76 and was found to display cytotoxicity against HEp-2 and HepG2 cells with IC_50_ values of 9 and 16 μmol/ml, respectively (Huang et al., 2013). In the assessment of a library of marine-derived fungi (240 strains) for growth inhibitory activity against *Mycobacterium phlei* (*M. phlei*), the extract of the sponge-derived fungus *Metarhizium anisopliae* mxh-99 displayed promising levels of anti-*M. phlei* activity and produced two naphtho-γ-pyrones glycosides, indigotides G–H (**147**–**148**, Figure 11; Kong et al., 2013).

#### Esters

The 3,4-dihydroisocoumarin derivative, R-(–)-mellein-8-*O*-β-D-glucopyranoside (**149**, Figure 11), was yielded from the marine-derived fungus *Paraconiothyrium sporulosum* YK-03 (Zhang et al., 2017). Another two isocoumarin glucosides, halorosellins A–B (**150**–**151**, Figure 11), were produced from a culture broth of the marine fungus, *Halorosellinia oceanica 187q* BCC 5149 (Chinworrungsee et al., 2002). Based on bioassay test, another two isocoumarin derivatives, acremonones H (**152**), and penicimarins D (**153**, Figure 11), were produced from the mangrove-derived fungus *Acremonium* sp. PSU-MA70 and the sponge-derived fungus, *Penicillium* sp. MWZ14-4, respectively (Rukachaisirikul et al., 2012; Qi et al., 2013). One mycophenolic acid derivative, penicacid B (**154**, Figure 11), was given by a fungus, *Penicillium* sp. SOF07 derived from a marine sediment in the South China Sea and was found to inhibit inosine-monophosphate dehydrogenase (IMPDH) (Chen et al., 2012). The marine-derived fungus *Cosmospora* sp. SF-5060, isolated from inter-tidal sediment, produced the depside derivative, aquastatin A (**155**, Figure 11). Aquastatin A was found to display potent selective inhibitory activity against protein tyrosine phosphatase 1B (PTP1B) with an IC_50_ value of 0.19 μM in a competitive manner (Seo et al., 2009). Another *C*-glycosidic depside, stromemycin (**156**, Figure 11), was identified from the crude extract of the fungus *Emericella variecolor* derived from the marine sponge *Haliclona valliculata* (Bringmann et al., 2003).

#### Lipids

**Figure 12:**
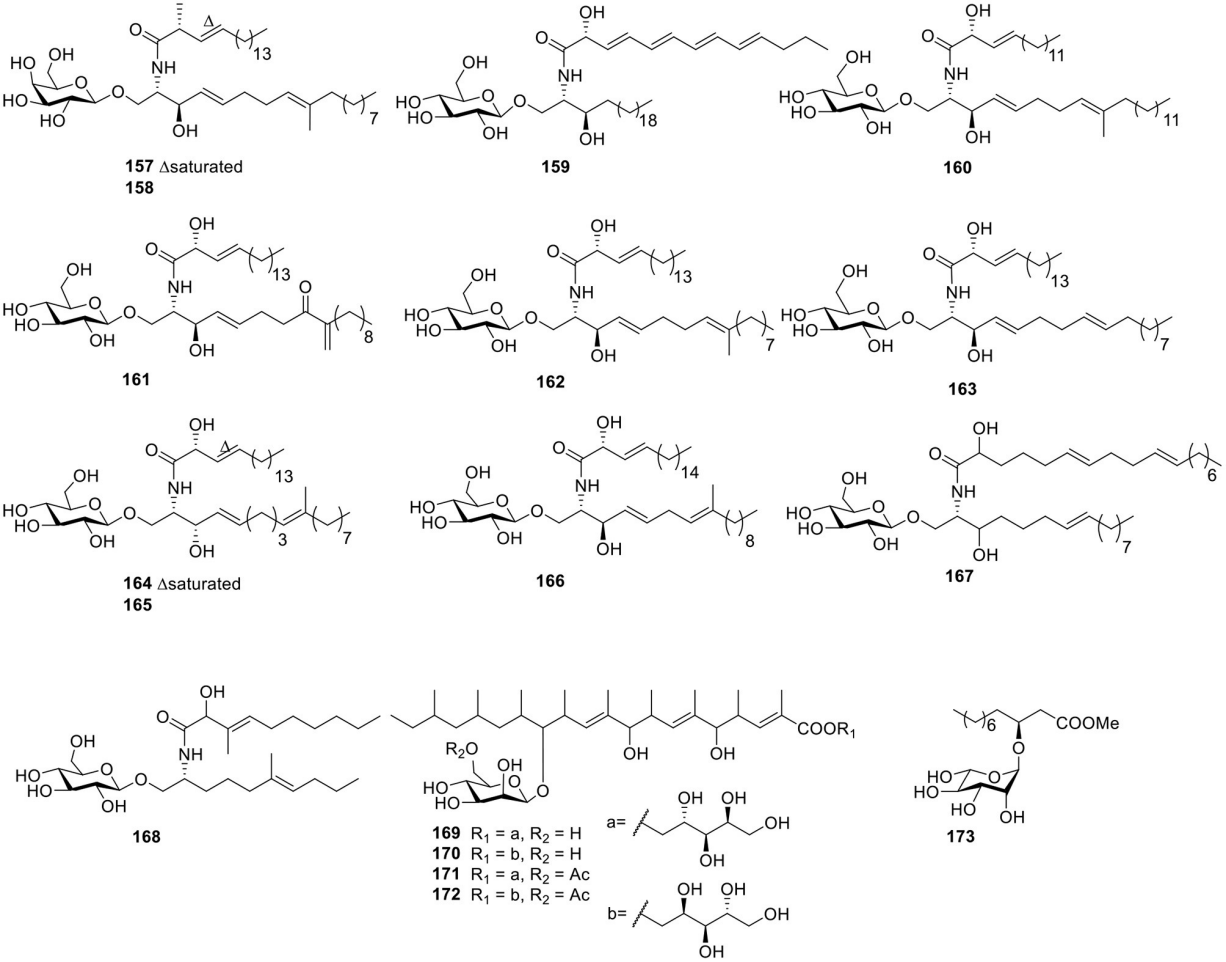
Chemical structures of compounds **157–173** derived from marine-sourced fungi.

Two cerebroside analogs, flavicerebrosides A and B (**157**–**158**, Figure 12), were obtained from the cultivated mycelium of the marine-derived fungus *Aspergillus flavipes*, isolated from the sea anemone *Anthopleura xanthogrammica* and exhibited cytotoxic activity against the KB cell line with IC_50_ values of 20.1 and 14.3 μg/ml, respectively (Jiang et al., 2004). The Quanzhou marine fungus *Aspergillus niger* (MF-16) produced another two cerebrosides, asperiamides B and C (**159**–**160**, Figure 12; Wu et al., 2008). With the constant effort to isolate microbes from hypersaline environments, the marine-derived halotolerant fungal strain (THW-18), *Alternaria raphani*, was isolated and afforded three cerebrosides, alternarosides A–C (**161**–**163**, Figure 12). Bioassay testing indicated that compounds **161**–**163** showed small antibacterial activity against *E. coli, B. subtilis*, and *C. albicans* (Wang et al., 2009). Flavusides A–B (**164**–**165**, Figure 12), two new antibacterial cerebroside derivatives, were identified from the fermentation broth of the marine-derived fungus *Aspergillus flavus* and showed weak inhibitory activities against *S. aureus* and methicillin-resistant *S. aureus* (Yang et al., 2011).

In order to explore bromodomains (BRD) inhibitors, the secondary metabolites of *Alternaria* sp. NH-F6, a fungus obtained from deep-sea sediment samples, were analyzed and led to a cerebroside (**166**, Figure 12) along with two perylenequinones (Ding et al., 2017). Under continuous searching for bioactive natural products from Red-Sea marine-derived fungi, the fungus *Penicillium* was isolated from the tunicate *Didemnum* and led to two cerebrosides, penicillosides A–B (**167**–**168**, Figure 12). In an agar diffusion assay, penicilloside A (**167**) exhibited antifungal activity against *C. albicans*, while penicilloside B (**168**) showed antibacterial activities against *S. aureus* and *E. coli* (Murshid et al., 2016). Four highly methylated glycolipids—roselipins 1A (**169**), 1B (**170**), 2A (**171**), and 2B (**172**, Figure 12)—were obtained from the marine fungus *Gliocladium roseum* KF-1040. In an enzyme assay system using rat liver microsomes, roselipins inhibited the enzyme, diacylglycerol acyl transferase (DGAT) with IC_50_ values of 17–22 μM (Omura et al., 1999; Tabata et al., 1999). One fatty acid, glucoside (**173**, Figure 12), was isolated from the endophytic fungus A1 of mangrove plant, *Scyphiphora hydrophyllacea Gaertn. F*. Through using a filter-paper disc-agar-diffusion method, compound **173** possessed modest inhibitory activity on *Staphylococcus aureus* and methicillin-resistant *S. aureus* (Zeng et al., 2012).

#### Terpenoids

**Figure 13:**
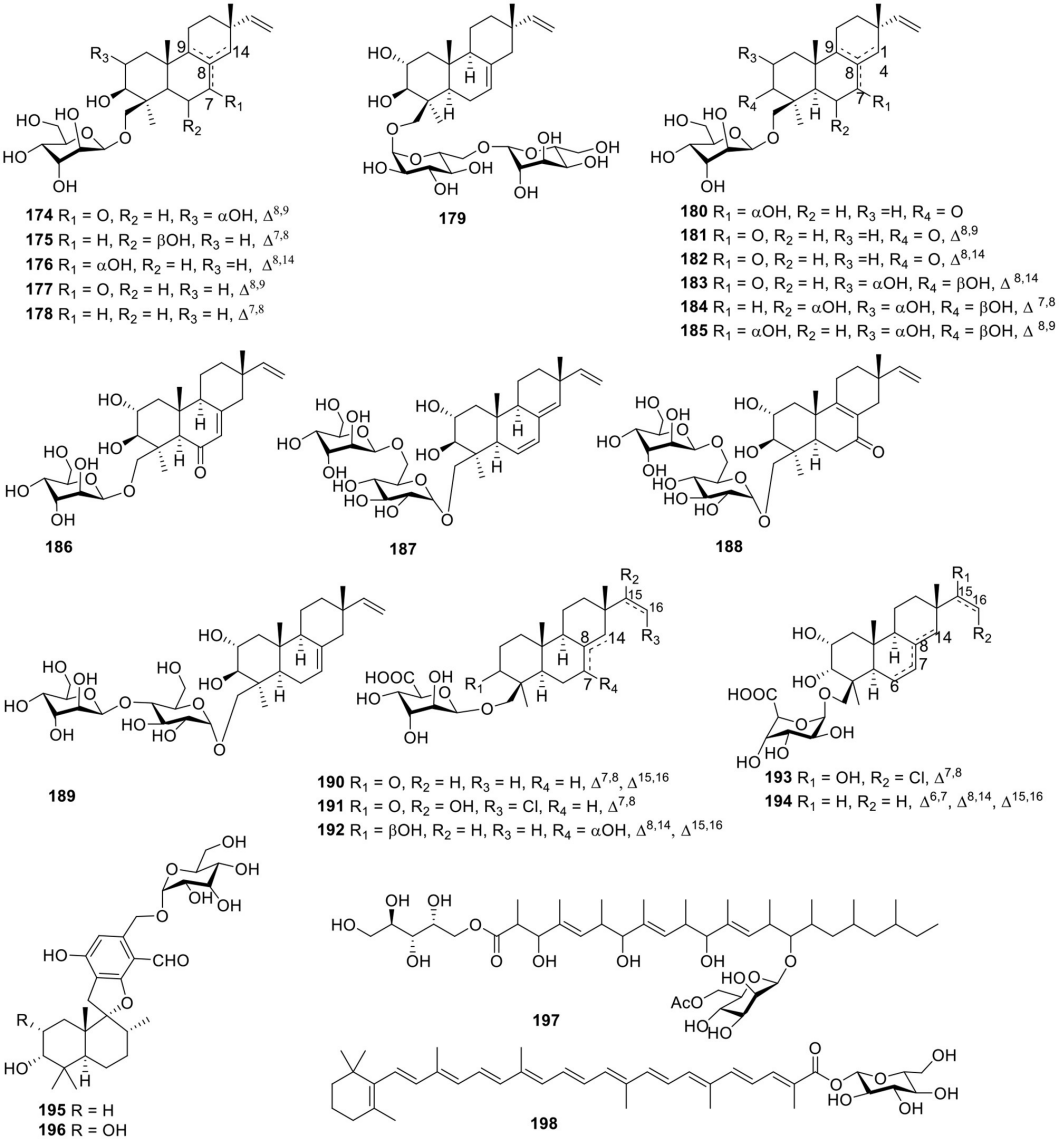
Chemical structures of compounds **174–198** derived from marine-sourced fungi.

The fungus *Acremonium striatisporum* was originally obtained from the holothurian *Eupentacta fraudatrix*, and multiple investigations for metabolites of this strain led to the isolation of 21 glycosides, namely virescenosides M–X (**174**–**185**), Z (**186**), R_1_-R_3_ (**187**–**189**), and Z_4_-Z_8_ (**190**–**194**, Figure 13). Bioassay testing showed that virescenosides M–U (**174**–**182**) displayed cytotoxic action against tumor cells Ehrlich carcinoma (IC_50_ = 10–100 μM) *in vitro*. In addition, virescenosides M–N (**174**–**175**) and P (**177**) displayed cytotoxic effects on developing eggs of the sea urchin, *Strongylocentrotus intermedius* (IC_50_ = 2.7–20 μM) (Afiyatullov et al., 2000, 2002, 2004, 2006, 2011, 2016). Two phenylspirodrimane-type glucosidic meroterpenoids, stachybosides A–B (**195**–**196**, Figure 13), were seperated from the sponge-derived fungus, *Stachybotrys chartarum* MXH-X73 (Ma et al., 2013). The polyketide glycoside, cladionol A (**197**, Figure 13), was produced from the culture broth of the fungus *Gliocladium* sp. L049 collected from the sea grass *Syringodium isoetifolium* and was found to exhibit modest cytotoxicity against murine leukemia L1210 cells and human epidermoid carcinoma KB cells with IC_50_ values of 5 and 7 μg/ml, respectively (Kasai et al., 2005). The carotenoid glycosyl ester, neurosporaxanthin β-D-glucopyranoside (**198**, Figure 13), was isolated from cultured cells of the marine microorganism, *Fusarium* sp., collected from the seawater surface and was the first naturally occurring neurosporaxanthin glycoside that was discovered (Sakaki et al., 2002).

#### Other Classes

**Figure 14:**
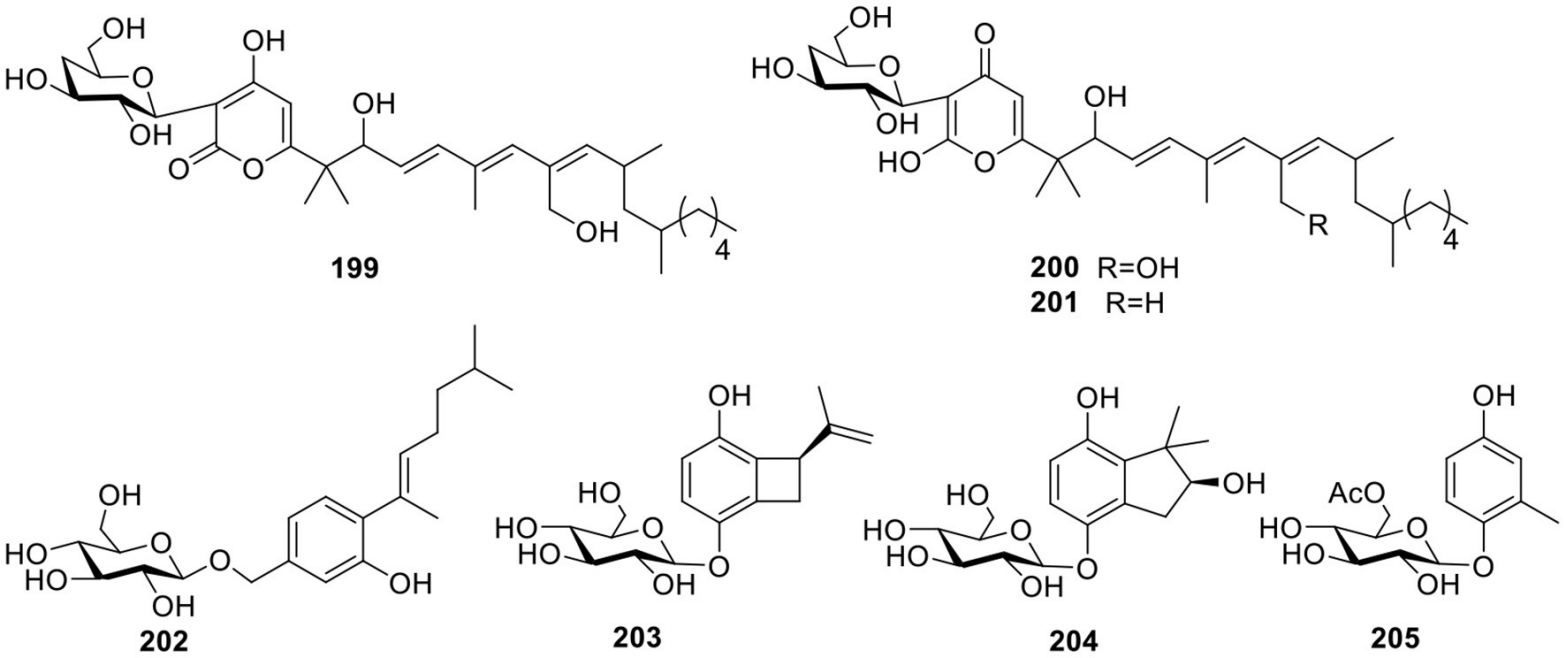
Chemical structures of compounds **199–205** derived from marine-sourced fungi.

Three polyketide glucosides containing a pyrone ring—neofusapyrone (**199**), fusapyrone (**200**), and deoxyfusapyrone (**201**, Figure 14)—were obtained from the marine-derived fungus *Fusarium* sp. FH-146. All three compounds exhibited moderate active against *Aspergillus clavatu* with MIC values of 6.25, 25, and 3.12 μg/ml, respectively (Hiramatsu et al., 2006). The fungus *Aspergillus sydowii* derived from the marine sponge, *Stelletta* sp., produced the β-D-glucopyranosyl, aspergillusene A (**202**, Figure 14), which was the first glycoside of phenolic bisabolane sesquiterpenes that was discovered and exhibited mild antitumor activities against KB, HepG2 and HCT-116 cell lines (Liu et al., 2017). Three hydroquinone glycosides—acremonin A1-*O*-β-D-glucopyranoside (**203**), gliomastin E1-O-β-D-glucopyranoside (**204**), and 6′-*O*-acetyl-isohomoarbutin (**205**, Figure 14)—were isolated from the marine-derived fungus, *Gliomastix* sp., originally derived from the hard coral, *Stylophora* sp. (Elnaggar et al., 2017).

## Conclusions

**Figure 15:**
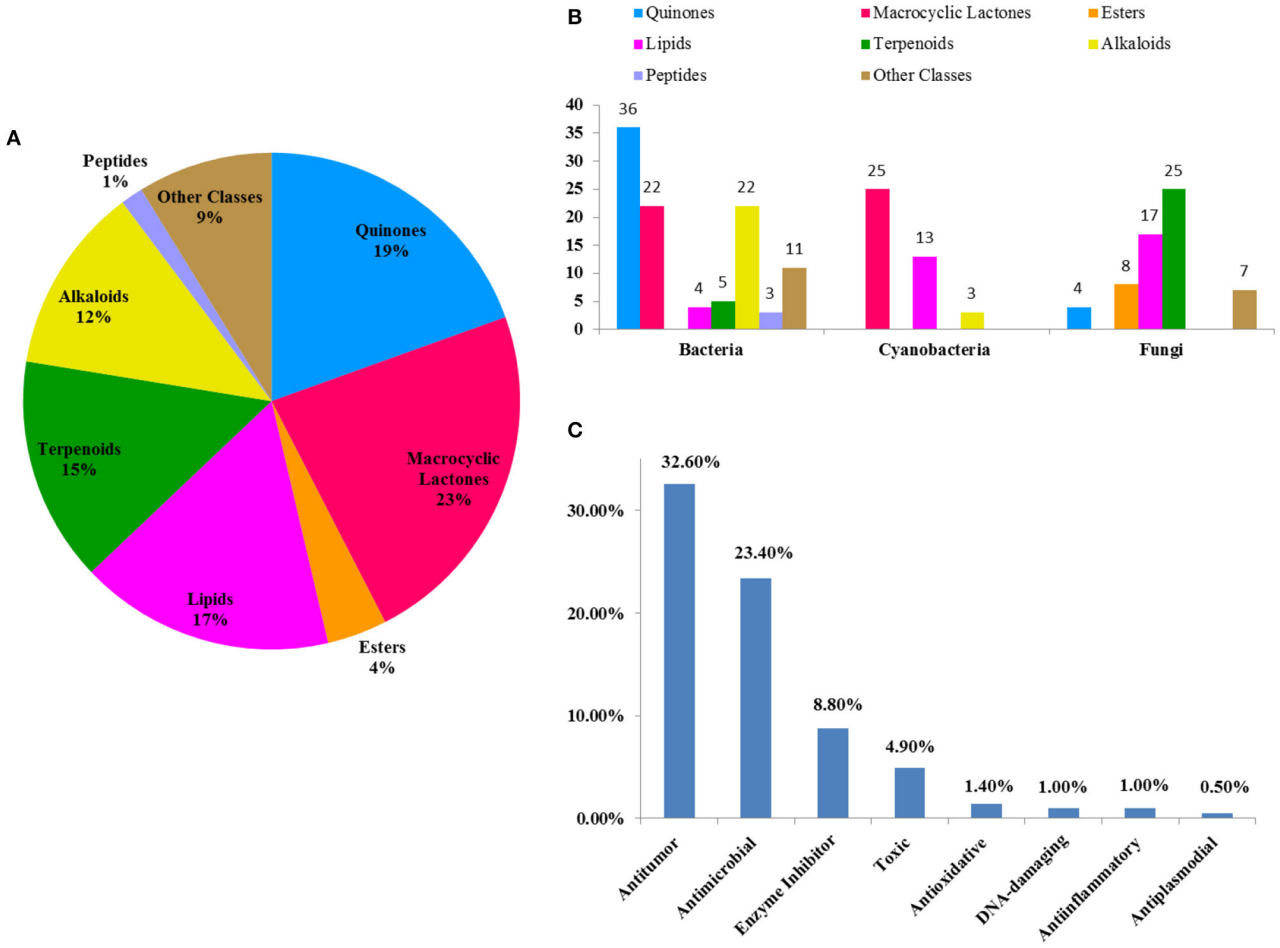
The percent of each class glycosides out of all glycosides **(A)**; the distribution of each class glycosides in marine microbes **(B)**; the percent of each class bioactive glycosides out of all glycosides **(C)**.

According to an estimation, ~70% of global drug leads derive directly from natural products, many of which are glycosylated metabolites (Thorson et al., 2001). Chemical investigation for 205 glycosides of the last 22 years (1997–2018) suggests that these compounds are classified as quinones, macrocyclic lactones, esters, lipids, terpenoids, alkaloids, peptides, and other classes. Macrocyclic lactones and quinone glycosides comprise roughly 42% of all these compounds (Figure 15A), and bacteria were the main source of new glycosides at 50% (104/205) (Figure 15B). Given the importance of glycoprotein to many biological processes, although peptide glycosides only account for 1% of these compounds, the peptide glycosides have a considerable potential for the discovery of drug leads.

In this review, the bioactivities of 129 glycosides were summarized in Tables S1–S3. In measured activities for these compounds, more than 50% (Figure 15C) display antitumor and antimicrobial activities, some of which also possess strong cytotoxicity. For example, IB-00208 (**16**) had a strong antibiotic activity against Gram-positive organisms with MIC values ranging from 0.09 to 1.4 nM, and lomaiviticin A (**34**) had a unique cytotoxicity profile against cancer cell lines with IC_50_ values ranging from 0.01 to 98 ng/ml. At present, two FDA-approved marine drugs, *ara*-C and *ara*-A, are antitumor and antiviral nucleosides, which are consistent with the main activity summarized in this review. This suggests that antitumor and antimicrobial drugs may be the main research direction for marine natural products. In addition, some glycosides also exhibited enzyme-inhibitory, antioxidative, DNA-damaging, anti-inflammatory, and anti-plasmodial activities. The recent in-depth study of glycosides has revealed their dynamic potential as therapeutic agents in the treatment of different disorders. Based on these findings, it may be possible to discover and develop glycosides with higher selectivities and efficacies.

## Author Contributions

KL and XZ designed and elaborated the manuscript. JC, ZS, BY, XZ, and YL added valuable comments. XZ, JH, and HT critically revised and improved the manuscript. All authors read and approved the final version of the manuscript.

### Conflict of Interest

The authors declare that the research was conducted in the absence of any commercial or financial relationships that could be construed as a potential conflict of interest.
