# Supplementary material for: Glycosylated Natural Products From Marine Microbes
Source: Front Chem. 2020 Jan 10;7:879. doi: 10.3389/fchem.2019.00879 (PMC6965366; doi:10.3389/fchem.2019.00879)
Supplement: Supplementary file 1 [file Data_Sheet_1.pdf]

Supplementary Material

List of Supporting Information

Table S1. Compounds with significant activities from marine-sourced bacteria………………………………………………………………..2

Table S2. Compounds with significant activities from marine-sourced cyanobacteria……………………………………………………………….4

Table S3. Compounds with significant activities from marine-sourced fungi……………………………………………………………………...5

Table S1. Compounds with significant activities from marine-sourced bacteria

Chemical Type	Compounds	Activity
Quinones	**1–2**	against *Bacillus subtilis*
	**5**	against the neuroblastoma cell line (Neuro 2A) at 1 μg/ml
	**6–9**	against P388 cultured cells, ID_{50} = 0.4–0.06 μg/ml
	**11**	against the HeLa and HL-60 cells, IC_{50} =120 and 210 μM, respectively.
	**12–13**	against *Candida albicans* and *Xanthomonas* sp. pv. *Badrii*.
	**14**	against several human cancer cell lines, IC_{50} =0.15–0.33 μM<br>against a number of bacteria including antibiotic-resistant strains, IC_{50} = 0.31–31.2 μM
	**15**	against HCT-116 cells, IC_{50} = 1.16 μg/ml<br>against a panel of human Gram+ pathogens, MIC < 1 μg/ml
	**16**	against several lines of tumor cells, both human and murine<br>against Gram-positive organisms MIC = 0.09–1.4 nM
	**17**	against tumor cell lines SF-268 and MCF-7, IC_{50} = 6.70 and 8.02 μM, respectively.
	**19**	against the large cell lung cancer xenograft LXFL 529, IC_{50} = 0.26 ng/ml
	**22**	against *Bacillus subtilis* and methicillin-sensitive *Staphylococcus aureus* (MSSA), MIC = 8 and 2 μg/ml , respectively.
	**25–27**	against *Bacillus subtilis* at 4.1 μM, 4.2 μM and 4.3 μM, respectively
	**29**	against human cancer cell MCF10A, IC_{50} = 2.9 μM
	**30**	against human cancer cells MDA-MB-435, MDA-MB-231, NCI-H460, HCT-116, HepG2, and human normal breast epithelial cell MCF10A, IC_{50} = 2.6–5.4 μM
	**32–33**	against *Pseudomonas aeruginosa*, MRSA,*Klebsiella pneumonia*, and *Escherchia coli*, MIC = 16 μg/ml
	**34**	potent DNA-damaging agent, ≤ 0.1 ng/spot<br>against a number of cancer cell lines, IC_{50} = 0.01–98 ng/ml<br>against *Staphylococcus aureus* and *Enterococcus faecium*
	**35**	potent DNA-damaging agent, ≤ 0.1 ng/spot<br>against *Staphylococcus aureus* and *Enterococcus faecium*
Macrocyclic Lactones	**37**	against a variety of microorganisms and microalgae
	**41–43**	against *Staphylococcus aureus* 209P, MIC = 16–32 μg/ml
	**45**	against six cell lines of glioma C6, U87-MG, SHG-44, U251 and colorectal cancer HCT-15 and SW620, IC_{50} = 20.84–81.01 μM.
	**46**	against the murine P388 cell line

	**47**	against *Bacillus subtilis* (KCTC 1021), *Staphylococcus aureus* (KCTC 1916), *Escherichia coli* (KCTC 1923) and *Pseudomonas aeruginosa* (KCTC 2592), MIC = 64 μg/ml
	**48–49**	potent anti-inflammatory activities in the PMA -induced mouse ear edema model
	**50**	against the cell proliferation of 7402 hepatoma cells, IC_{50}= 0.6 μg/ml
	**51**	against the human breast cancer cell line MDA-MB 435, IC_{50} = 7.5 μM.
	**52–54**	against *Bacillus subtilis* BS01 and *Bacillus thuringiensis* BT01, MIC = 0.016–0.5 μg/ml
	**55–58**	against *Bacillus subtilis* BS01 and *Bacillus thuringiensis* BT01, MIC = 1.0–8.0 μg/ml
Lipids	**60**	against lung cancer (NCI-H23) and stomach cancer (NUGC-3) cell lines, GI_{50} = 25.18, and 17.78 μg/ml, respectively
	**61**	against *Burkholderia cenocepacia* and *Staphylococcus aure*i, MBC= 3.12 μg/ml and 3.12 μg/ml, respectively
Terpenoids	**63**	potent antioxidative activity, IC_{50} = 4.6 μM (10.9 μM for β-carotene)
	**64**	$^1O_2$ suppression activity, IC_{50} = 5.1 μM
	**67**	$^1O_2$ suppression activity, IC_{50} = 5.1 μM
Alkaloids	**68–79**	against PC-3 cell lines, IC_{50} = 0.8–41.3 μM<br>potent kinase inhibitory activity against PKCα, ROCK2, BTK and AKS1
	**80**	against *Bacillus subtilis*
	**82**	against *Plasmodium falciparum* lines Dd2 and 3D7, IC_{50} = 5.03 and 10.43 μM, respectively
	**84**	against *Bacillus thuringiensis*, *Bacillus thuringiensis*, and *Candida albicans*, MIC = 2.0, 0.5 and 8.0 μg/ml, respectively.
	**85**	against methicillin-resistant *Staphylococcus aureus*, *Escherichia coli*, and *Candida albicans* MIC = 27.0-37.0 μg/ml
	**86**	against three multidrug resistance (MDR) and drug-sensitive parental cell lines
	**87**	reverse the multidrug resistance of K562/A02,MCF-7/Adr, and KB/VCR cells at 10 μM
	**88**	against three multidrug resistance (MDR) and drug-sensitive parental cell lines
Peptides	**90–91**	against a broad spectrum of Gram-positive bacteria, MIC = 0.1-60 ng/ml
Other Classes	**96**	against different human tumour cell line, such as MiaPaca_2, MCF-7 and HepG2, IC_{50} = 2.70, 1.58 and 4.30 μM, respectively
	**97–98**	against three human tumor cell lines, GI_{50} = 0.24–2.69 μM
	**99**	against a panel of 36 human tumor cell lines with concentration-dependent cytotoxicity, IC_{50}= 2.0 μM
	**103**	quinone reductase induction activity at 20 μM

Table S2. Compounds with significant activities from marine-sourced cyanobacteria

Chemical Type	Compounds	Activity
Macrocyclic Lactones	**105**	against KB cells, IC_{50} = 4.3 μM<br>against LoVo cells, $IC_{50} \approx 15$ μM
	**106**	against neuro-2a neuroblastoma cells, IC_{50} = 17 μM
	**110**	against a human tumor cell line panel
	**111**	induce apoptosis in HeLa S_3 cells and HL60 cells
	**114**	reduce IL-2 production without significantly affecting cell viability
	**115-116**	against HeLa S_3 cells, IC_{50} = 0.1 and 0.16 μM, respectively
	**117**	against the human lung carcinoma cell line H-460, EC_{50} = 2.5 μM
	**118**	against in NCI-H460, Neuro-2a, and MDA-MB-435 cell lines, IC_{50} =119 nM, 262 nM and 8.9 nM, respectively
	**120-128**	against the human lung cancer cell line H-460, IC_{50} = 170- 910 nM
Lipids	**129**	a toxic for brine shrimp and fish
	**134**	against the MG-63, RKO and T-47D cancer cell lines, IC_{50} = 39, 40 and 22 μM, respectively
	**141**	against MCF-7 cancer cell line, IC_{50} = 20.5 μM
Alkaloids	**143-144**	against MCF-7 cancer cell line, IC_{50} = 18.2 and 22.8 μM, respectively

Table S3. Compounds with significant activities from marine-sourced fungi

Chemical Type	Compounds	Activity
Quinones	**145**	against DPPH, IC_{50} = 14.2 μM<br>against the methicillin-resistant and multidrug-resistant *Staphylococcus aureus*, MIC = 50.0 μg/ml
	**146**	against HEp-2 and HepG2 cells, IC_{50} = 9 and 16 μmol/ml, respectively
Esters	**154**	an inhibitor for inosine-monophosphate dehydrogenase (IMPDH)
	**155**	against a protein tyrosine phosphatase 1B (PTP1B), IC_{50}= 0.19 μM
	**157**	against murine leukemia L1210 and human epidermoid carcinoma KB cells, IC_{50} =5 and 7 μg/ml, respectively.
Lipids	**158–159**	against the KB cell line, IC_{50} = 20.1 and 14.3 μg/ml, respectively
	**162–164**	against *Escherichia coli*, *Bacillus subtilis*, and *Candida albicans*
	**165–166**	against *Staphylococcus aureus* and methicillin-resistant *Staphylococcus aureus*
	**168**	against *Candida albicans*
	**169**	against *Staphylococcus aureus* and *Escherichia coli*
	**170–173**	inhibitors for the enzyme diacylglycerol acyl transferase (DGAT), IC_{50} = 17–22 μM
	**174**	against *Saphylococcus aureus* and methicillin-resistant *Staphylococcus aureus*.
Terpenoids	**175–176**	against tumor cells Ehrlich carcinoma, IC_{50} = 10–100 μM<br>cytotoxicity for developing eggs of the sea urchin *Strongylocentrotus intermedius*, IC_{50} = 2.7–20 μM
	**177–179**	against tumor cells Ehrlich carcinoma, IC_{50} = 20–100 μM<br>cytotoxicity for developing eggs of the sea urchin *Strongylocentrotus intermedius*
	**180–183**	against tumor cells Ehrlich carcinoma, IC_{50} = 25–60 μM<br>cytotoxicity for developing eggs of the sea urchin *Strongylocentrotus intermedius*, IC_{50} = 100–150 μM
Other Classes	**199–201**	against *Aspergillus clavatu*, MIC= 6.25, 25 and 3.12 μg/ml, respectively.
	**202**	against KB, HepG2 and HCT 116 cancer cell lines

.
